# Preparation and Characterization of Plant Protein Adhesives with Strong Bonding Strength and Water Resistance

**DOI:** 10.3390/foods11182839

**Published:** 2022-09-14

**Authors:** Yang Qu, Qin Guo, Xuegang Huang, Tian Li, Manzhu Liang, Jingjing Qin, Qiang Gao, Hongzhi Liu, Qiang Wang

**Affiliations:** 1 Institute of Food Science and Technology, Chinese Academy of Agricultural Sciences/Key Laboratory of Agro-Products Processing, Ministry of Agriculture, Beijing 100194, China; 2 Beijing Key Laboratory of Wood Science and Engineering, Beijing Forestry University, Beijing 100083, China

**Keywords:** byproducts, renewable resources, protein adhesive, water resistance, bonding strength

## Abstract

Plant protein adhesive has received considerable attention because of their renewable raw material and no harmful substances such as formaldehyde. However, for the plant protein adhesive used in the field of plywood, low cost, strong water resistance, and high bonding strength were the necessary conditions for practical application. In this work, a double-network structure including hydrogen bonds and covalent bonds was built in hot-pressed peanut meal (HPM) protein (HPMP) adhesive, soybean meal (SBM) protein (SBMP) adhesive and cottonseed meal (CSM) protein (CSMP) adhesives. The ether bonds and ester bonds were the most in CSMP adhesive, followed by SBMP adhesive, while the hydrogen bond was the most in HPMP adhesive. The solubility of the HPMP, SBMP, and CSMP adhesives decreased by 14.3%, 24.2%, and 19.4%, the swelling rate decreased by 56.9%, 48.4%, and 78.5%, respectively. The boiling water strength (BWS) of HPMP (0.82 MPa), SBMP (0.92 MPa), and CSMP adhesives reached the bonding strength requirement of China National Standards class I plywood (type I, 0.7 MPa). The wet shear strength (WSS) of HPMP, SBMP, and CSMP adhesives increased by 334.5% (1.26 MPa), 246.3% (1.42 MPa), and 174.1% (1.59 MPa), respectively. This study provided a new theory and method for the development of eco-friendly plant meal protein adhesive and promotes the development of green adhesive.

## 1. Introduction

Trialdehyde adhesive (urea formaldehyde resin, melamine formaldehyde resin, and phenolic resin) was widely used in the traditional wood industry [1]. However, trialdehyde adhesive had some problems, such as relying on oil resources, releasing formaldehyde, and polluting the environment [2]. Especially with the rise of global oil prices and the enhancement of people’s awareness of environmental protection and safety, the development of renewable biomass aldehyde-free adhesives (starch adhesives [3], tannin adhesives [4], lignin adhesives [5], plant protein adhesives [6]) have become an inevitable trend in the development of adhesive industry.

Soybean meal (SBM) as a widely sourced, environmentally friendly, and renewable biomass adhesive material has attracted much attention in recent years [7]. SBM has been widely studied in the area of plant protein adhesive, with the treatment reagents including enzyme [8], surfactants [9], urea [10], alkali [11], and epoxy resin [12]. The results showed that the dry shear strength (DSS) of SBM protein adhesive was close to that of formaldehyde adhesive, but the water resistance and wet shear strength (WSS) were poor due to large amounts of hydrophilic groups on the surface of soybean protein. Thus, the current research mainly focuses on how to improve the water resistance and bonding strength. At present, the commonly methods include the modification of phytic acid and aminoclay-cellulose nanofiber [13], the crosslinking of glyoxal and carboxylic acid [14], and catechol/polyamine and pyrogallol/polyamine co-deposition [15]. Although they improved water resistance, these methods were costly and only met the China National Standards class II plywood (type II).

Multiple network-enhancement strategies could improve the properties of materials [16]. The literature reported that the introduction of hydrophobic or non-zwitterionic polyelectrolyte components or metal coordination bonds to form a three-network structure could improve the mechanical strength of zwitterionic hydrogels in saline environment [17]. An organic–inorganic adhesive with three-level interpenetrating network structure was obtained based on the crosslinking and synergistic interaction between triglyceride, waterglass and polyaryl polymethylene isocyanate ripolyglycerol, and waterglass and polyaryl polymethylene isocyanate [18]. Soybean protein was hydrolyzed into peptides by bromelase, and then, a close triple-network structure was constructed with 1,2,3-propanetriol-diglycidyl-ether, tannic acid, and Zn^2+^ [19]. Notably, they all involved complex crosslinking reactions and chemical modification, which made them expensive and time-consuming, and could not meet the requirements of practical application. Therefore, it was still a challenge to build a cost-effective network structure that could significantly improve water resistance and bonding strength [20].

In this study, we aimed to design a simple and effective hydrogen-covalent double network to overcome the above challenges. Firstly, the reactivity of protein was improved by surfactant, and the first-level network was formed through hydrogen bonding. On this basis, the second-level network (covalent bond) was formed to improve the crosslinking density. The prepared hot-pressed peanut meal (HPMP), SBM, and cottonseed meal (CSM) adhesives had strong water resistance and bonding strength.

## 2. Materials and Methods

### 2.1. Materials

This research was conducted in Beijing from September to October 2021.

HPM (300 mesh, 48.6% protein, 6.74% moisture content, 7.11% ash, 4.36% fat, 27.3% carbohydrates, and 5.89% fiber) was provided by Shandong JinSheng grain, oil and Food Co., Ltd. (Linyi, Shandong, China); SBM (300 mesh, 46.6% protein, 7.46% moisture content, 9.05% ash, 10.05% fat, 20.09% carbohydrates, and 6.75% fiber) and CSM (300 mesh, 53.4% protein, 5.61% moisture content, 9.55% ash, 3.55% fat, 17.25% carbohydrates and 10.64% fiber) were provided Shandong YuHuang grain, oil and Food Co., Ltd. (Linyi, Shandong, China) and ChenGuang Oil Co., Ltd. (Shijiazhuang, Hebei, China), respectively. SDS and nSiO_2_ were purchased from Shanghai Macklin Biochemical Co., Ltd. (Shanghai, China). Polyamide epichlorohydrin resin (PAE, 25 wt%) was purchased from Zhejiang ChuanHua Huayang Chemical Co., Ltd. (Hangzhou, Zhejiang, China). Poplar veneer (30 × 30 × 0.18 in, 8.7% moisture content) was obtained from Wenan, Hebei province, China.

### 2.2. Preparation of HPM, SBM and CSM Protein Adhesives

**Figure 1:**
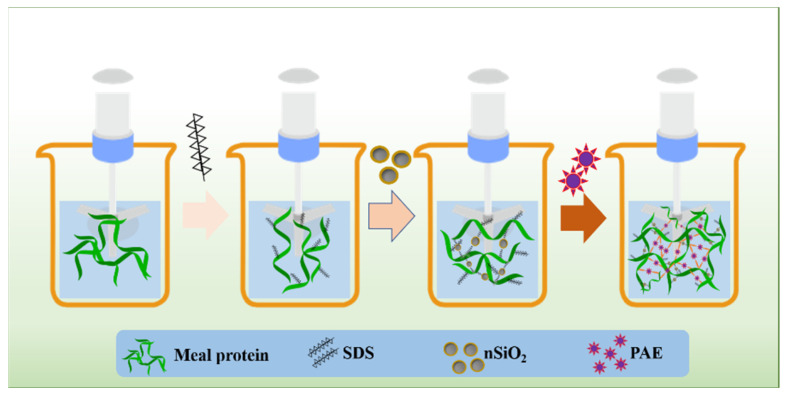
Scheme of the preparation of HPMP-, SBMP-, and CSMP-based adhesive.

The preparation methods of HPM protein (HPMP), SBM protein (SBMP), and CSM protein (SBMP)-based adhesives were prepared according to Qu et al. [21] and appropriately improved. The specific steps were as follows (Figure 1 and Table 1).

We added 40 g HPM, SBM, and CSM powder (300 mesh) into 120 g distilled water and stirred for 30 min to prepare pure HPMP, SBMP, and CSMP adhesives. SDS (1.28 g) was added to pure HPMP, SBMP, and CSMP adhesives and stirred for 30 min to obtain SDS/HPMP, SDS/SBMP, SDS/CSMP adhesives, respectively. nSiO_2_ (0.8 g) was introduced into SDS/HPMP, SDS/SBMP, and SDS/CSMP adhesives and stirred for 30 min to obtain SDS/nSiO_2_/HPMP, SDS/nSiO_2_/SBMP, and SDS/nSiO_2_/CSMP adhesives, respectively. HPMP, SBMP, and CSMP adhesives could be prepared by mixing PAE (34 g) with SDS/nSiO_2_/HPMP, SDS/nSiO_2_/SBMP, and SDS/nSiO_2_/CSMP adhesives for 30 min.

### 2.3. Characterization of Adhesive Samples

#### 2.3.1. Fourier-Transform Infrared Spectroscopy (FTIR)

The FTIR spectrometer (Tensor 27, Bruker) of the cured adhesive samples (200 mesh) were used to collect in the wavelength range between 400–4000 cm^−1^ with a resolution of 4 cm^−1^ with 64 scans [18].

#### 2.3.2. X-ray Photoelectron Spectroscopy (XPS)

The XPS (Thermo Fisher Scientific, Waltham, MA, USA) was used for monochromatic radiation detection of adhesive samples at ambient temperature [21].

#### 2.3.3. X-ray Diffraction (XRD)

The XRD analysis was performed using a commercial X-ray diffractometer (D8 Advance diffractometer, Bruker) ranging from 10° to 90° [16].

#### 2.3.4. Thermogravimetric (TGA)

The thermal gravimetric (TG) and differential thermogravimetric (DTG) curves of adhesive samples were obtained from TG analysis (Perkin Elmer) with a heating rate of 10 mL/min under nitrogen atmosphere [12].

#### 2.3.5. Scanning Electron Microscope (SEM)

All adhesive samples on the surface of the study were sprayed with gold, and the micro morphology of the cured adhesive samples was observed by SEM (Hitachi SU8010) [13].

### 2.4. Sol–Gel Test

The method was carried out according to Gao et al. [22]. The adhesive samples were dried in an oven at 110 °C to constant weight and then ground into powder (100 mesh) with a ball mill. The volume (*V_a_*) of 2.20 g (*M_a_*) of adhesive sample powder was recorded by the graduated cylinder, and then, the sample powder was heated in boiling water for 3 h. After filtration with a filter screen, the volume (*V_b_*) of the solid residue was measured again, and the residue was then dried at 110 °C until constant weight (*M_b_*). The solubility (Equation (1)) and swelling rate (Equation (2)) of the cured adhesive were as follows:

Solubility = (*M_a_* − *M_b_*)/*M_a_ ×* 100% (1)

Swelling ration = (*V_a_* − *V_b_*)/*V_a_ ×* 100% (2)

### 2.5. Preparation of Three-Ply Plywood Specimens and Bonding Strength Testing

Three-ply plywood was prepared with prepared HPMP, SBMP, and CSMP adhesives. The adhesive was evenly applied on the surface of poplar veneer with the amount of 220 g/m^2^. We manually assembled the three-layer veneer with vertical grain and then pressed it for 8 min under the conditions of 120 °C and 1.20 MPa. The prepared plywood was cut after being placed at room temperature for 24 h.

**Scheme 1:**
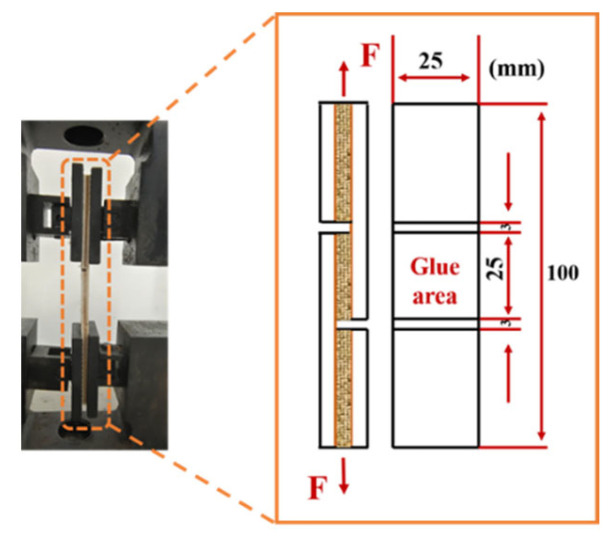
Plywood specimen size and tensile force loading diagram.

The boiling water strength (BWS, type Ⅰ), WSS (type Ⅱ), and DSS were tested at the speed of 5 mm/min by using a tensile testing machine according to the description of China National Standards GB/T 9846 (2015) [23]. The plywood panels as shown in Scheme 1. The plywood panels were soaked in boiling water for 4 h, dried at 63 °C for 20 h, then soaked in boiling water for 4 h and soaked in 25 °C water for 1 h, and dried at room temperature for 15 min, and the boiling water strength was tested. For WSS, the plywood panels were soaked in 63 °C water for 3 h, then aged at room temperature for 15 min and tested. All experiments were repeated three times, with 6 samples per group.

### 2.6. Physical Property Measurements of Adhesive Sample

Next, we put the fresh adhesive sample (*M_d_*) into an aluminum box and dried it to constant weight at 120 °C and weighed the mass of dry adhesive (*M_a_*). The calculation of solids content (Equation (3)) is presented in the following equation. The viscosity of adhesive samples was evaluated using a Brookfield DV-Ⅲ rotary viscometer. A surface tension-contact angle meter (SL200KB) was used to test the water contact angle (WCA) and solid surface energy (SSE) for the adhesive samples [24].

Solids content = *M_a_*/*M_d_ ×* 100% (3)

### 2.7. Crack Observations

The different adhesive samples (6 g) were evenly coated on one side of the glass slide, dried at 110 °C for 3 h, and then placed in a dryer to cool to room temperature. The surface morphology of the cured adhesive was observed and photographed with Canon camera (700 d).

### 2.8. Statistical Analysis

The significant difference of bonding strength was analyzed by analysis of variance (ANOVA) test in SPSS 22.0. XRD, FTIR, and XPS spectra were drawn by origin 8.5.

## 3. Results and Discussion

### 3.1. Characterization of HPMP, SBMP, and CSMP-Based Adhesives

**Figure 2:**
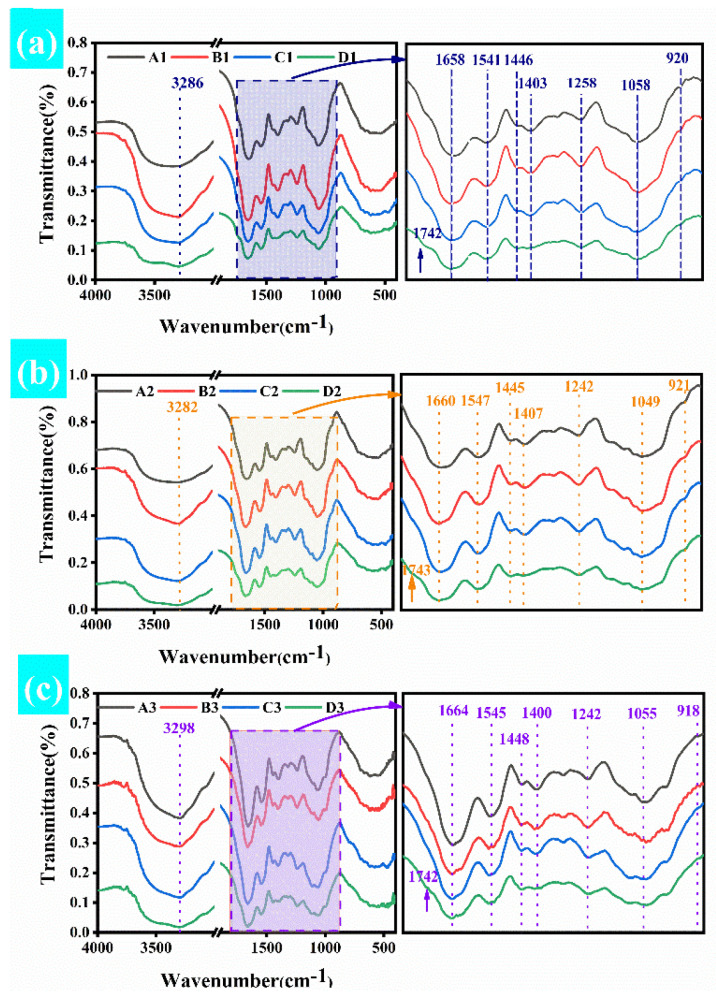
FTIR spectra of (**a**) A1-pure HPMP, B1-SDS/HPMP, C1- SDS/nSiO_2_/HPMP, and D1-HPMP adhesives. (**b**) A2-pure SBMP, B2-SDS/SBMP, C2-SDS/nSiO_2_/SBMP, and D2-SBMP adhesives. (**c**) A3-pure CSMP, B3-SDS/CSMP, C3- SDS/nSiO_2_/CSMP, and D3-CSMP adhesives.

**Figure 3:**
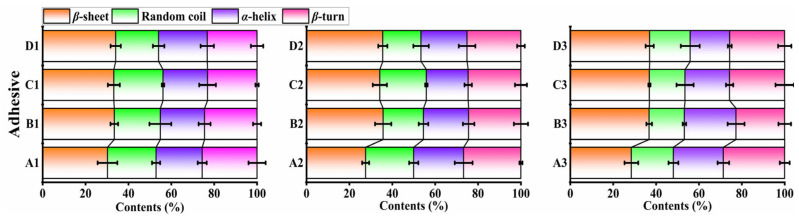
Secondary structure of A1-pure HPMP, B1-SDS/HPMP, C1-SDS/nSiO_2_/HPMP, D1-HPMP adhesives, A2-pure SBMP, B2-SDS/SBMP, C2-SDS/nSiO_2_/SBMP, D2-SBMP adhesives, A3-pure CSMP, B3-SDS/CSMP, C3-SDS/nSiO_2_/CSMP, and D3-CSMP adhesives.

As shown in the Figure 2, the pure HPMP, SBMP, and CSMP adhesives had obvious characteristic peaks at 1635–1637 cm^−1^, 1529–1533 cm^−1^, and 1238–1255 cm^−1^, representing C=O stretching (amide I, primary amine), N-H bending (amide II, secondary amine), and C-N and N-H stretching (amide III), respectively [22]. As could be seen from Figure 2a–c, compared with pure HPMP, SBMP, and CSMP adhesives, the peak areas of SDS/HPMP, SDS/SBMP, and SDS/CSMP adhesives at 1403 cm^−1^, 1400 cm^−1^, and 1407 cm^−1^, respectively, increased to varying degrees, indicating that the contents of -COOH increased. This was because SDS unfolds the protein structure and exposed more internal active groups. The results were consistent with the changes of secondary structure. The evidence showed the content of *β*-sheet increased, the peptide chain was more extended, and the reactivity of the adhesive increased (Figure 3). The addition of nSiO_2_ was an indispensable factor in the construction of the first-level network. The characteristic peak intensities of SDS/nSiO_2_/HPMP, SDS/nSiO_2_/SBMP, and SDS/nSiO_2_/CSMP adhesives at 918–921 cm^−1^, 1400–1406 cm^−1^, and 3282–3298 cm^−1^ were significantly reduced. Note that the hydrogen bonds formed between nSiO_2_ as well as O-H and N-H. After the introduction of PAE, the tertiary structure of protein was expanded by electrostatic and hydrophobic forces, which made it more flexible. The -COOH peak areas of HPMP, SBMP, and CSMP adhesives decreased significantly, and the new absorption peaks were observed at 1743 cm^−1^, 1722 cm^−1^, and 1720 cm^−1^, which was caused by the formation of ester bond between -OH and -COOH [25]. The C-O stretching vibration (ethanol and phenol) of HPMP, SBMP, and CSMP adhesives at 1060 cm^−1^, 1055 cm^−1^, and 1049 cm^−1^ was caused by the reaction of azacyclobutane with -OH to formed ether bond. The absorption peak intensities of HPMP, SBMP, and CSMP adhesives at 1529 cm^−1^, 1531 cm^−1^, and 1533 cm^−1^ decreased significantly, suggesting that the primary amine was also involved in the crosslinking reaction. In addition, their peaks at 3280–3298 cm^−1^ were the widest, indicating that the double-network structure formed by covalent bonds (ester bonds and ether bonds) and hydrogen bonds was dense.

**Figure 4:**
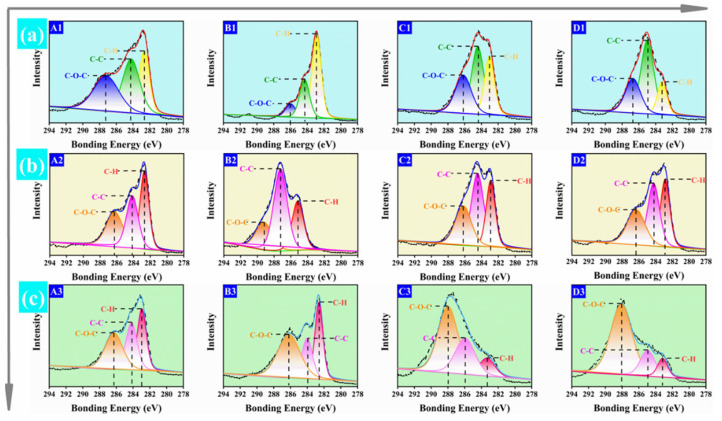
C 1s core-level spectra of (**a**) A1-pure HPMP, B1-SDS/HPMP, C1-SDS/nSiO_2_/HPMP, and D1-HPMP adhesives. (**b**) A2-pure SBMP, B2-SDS/SBMP, C2-SDS/nSiO_2_/SBMP, D2-SBMP adhesives. (**c**) A3-pure CSMP, B3-SDS/CSMP, C3-SDS/nSiO_2_/CSMP, and D3-CSMP adhesives.

**Figure 5:**
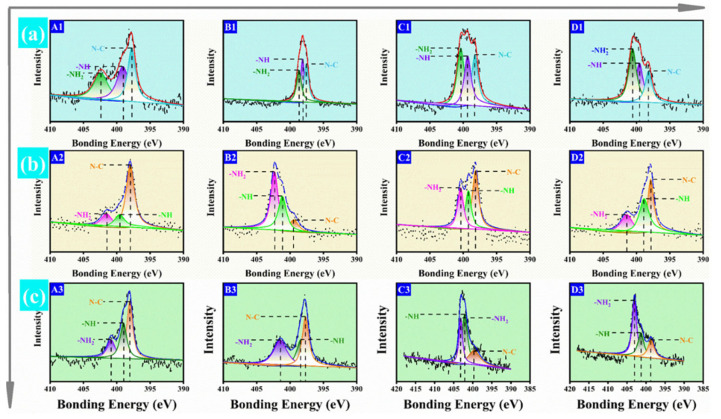
N 1s core-level spectra of (**a**) A1-pure HPMP, B1-SDS/HPMP, C1-SDS/nSiO_2_/HPMP, and D1-HPMP adhesives. (**b**) A2-pure SBMP, B2-SDS/SBMP, C2-SDS/nSiO_2_/SBMP, and D2-SBMP adhesives. (**c**) A3-pure CSMP, B3-SDS/CSMP, C3-SDS/nSiO_2_/CSMP, and D3-CSMP adhesives.

To better understand the compositional change and interior interactions in the adhesives by XPS (Figure S1), the binding energy and characteristic peaks [26] of different adhesive samples are shown in Figure 4 and Figure 5. Compared with pure HPMP, SBMP, and CSMP adhesives, the C 1s and N 1s core-level spectra of SDS/HPMP, SDS/SBMP, and SDS/CSMP adhesives increased in varying degrees, which proved that SDS contributed to more active group exposure. After the introduction of SiO_2_, the peak area of SDS/nSiO_2_/HPMP adhesive was the largest at 400.38 eV (Figure 5), indicating that the content of -NH_2_ was the most; the findings showed the most hydrogen bond formation in this case, while the peak area of SDS/nSiO_2_/SBMP adhesive was the smallest, and the hydrogen bonds were the fewest. The highest C-O-C content of CSMP adhesive was due to the highest peak strength at 288.09 eV, which was helpful to form more ether bonds and ester bonds. At the same time, from the peak strength of HPM adhesive at 200 eV, it could be seen that ether bond content was much lower than that of SBMP and CSMP adhesives. It was worth noting that CSMP adhesive moved to the left at 398.67 eV, indicating that PAE had more self-crosslinking, further improved its water resistance [27].

**Figure 6:**
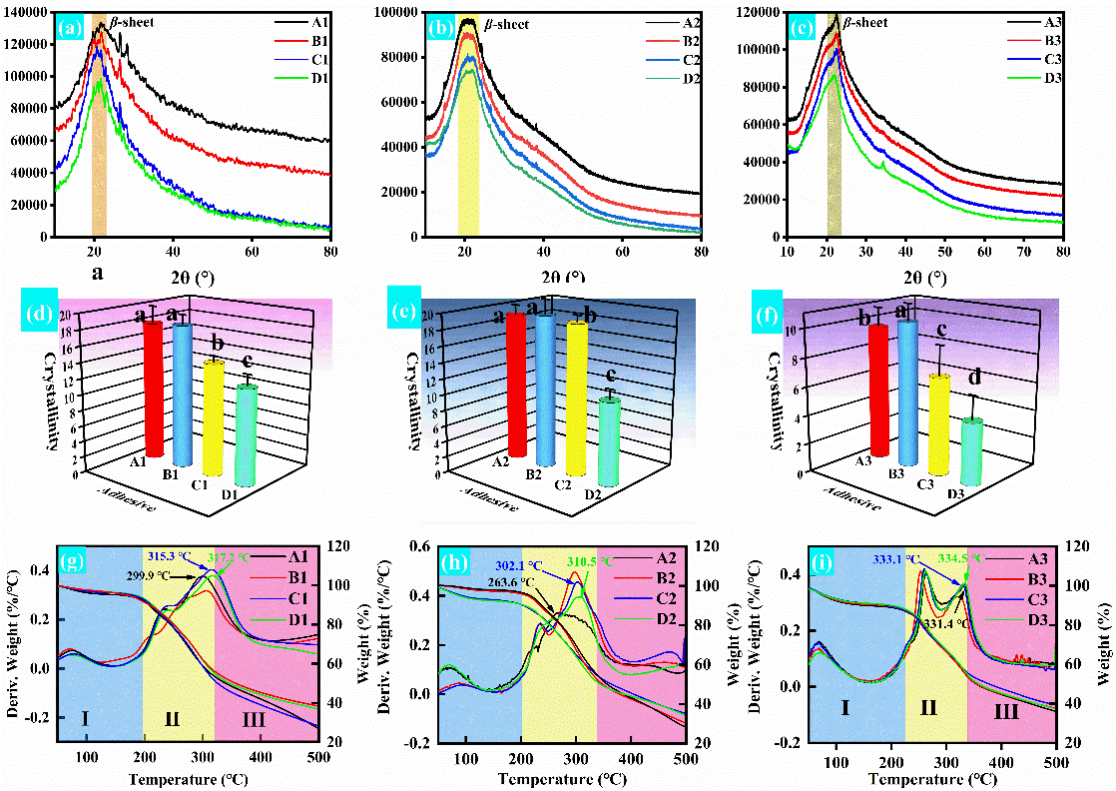
(**a**–**c**) XRD profiles, (**d**–**f**) crystallinity, (**g**–**i**) thermal degradation curves of A1-pure HPMP, B1-SDS/HPMP, C1-SDS/nSiO_2_/HPMP, D1-HPMP, A2-pure SBMP, B2-SDS/SBMP, C2-SDS/nSiO_2_/SBMP, D2-SBMP, A3-pure CSMP, B3-SDS/CSMP, C3-SDS/nSiO_2_/CSMP, and D3-CSMP adhesives.

The XRD patterns of different adhesives were shown in Figure 6a–c. Pure HPMP, SBMP, and CSMP adhesives had an obvious characteristic peak at 21.64°, 20.82°, and 22.68°, respectively (β-sheet) [28]. After the introduction of SDS, the crystallization peak areas of SDS/HPMP, SDS/SBMP, and SDS/CSMP adhesives increased in varying degrees, and the crystallinity increased by 6.3% (18.2°), 2.6% (19.4°), and 2.2% (10.2°), respectively. This was due to SDS destroying the structure of peanut protein, expanding the molecular structure, and being improved the crystallinity. For SDS/nSiO_2_/HPMP, SDS/nSiO_2_/SBMP, and SDS/nSiO_2_/CSMP adhesives, the peak strength of *β*-sheet decreased by 22.0% (14.2°), 2.1% (19.0°), and 33.3% (6.8°), respectively (Figure 6d–f), which confirmed that nSiO_2_ interacted with protein to form hydrogen bond. After the introduction of PAE, the crystallization peaks of HPMP, SBMP, and CSMP adhesives widened and moved to 21.66°, 21.91°, and 22.72°, and the crystallinity decreased significantly by 14.8% (12.1°), 45.3% (10.4°), and 36.8% (4.3°), which means that the crystal structure of protein molecules collapsed due to the crosslinking reaction, further improving the crosslinking density of the double network [29]. The crystallinity of CSMP adhesive was the lowest, and that of HPMP adhesive was the highest, indicating that the double network formed by CSMP adhesive was the densest. The results could be further proven by the thermal decomposition temperature. All adhesive samples had three thermal degradation stages. Different from HPMP- and SBMP-based adhesives, the CSMP-based adhesive had two degradation stages assigned to protein subunits with different stabilities [25]. The thermal decomposition temperatures of SDS/nSiO_2_/HPMP, SDS/nSiO_2_/SBMP, and SDS/nSiO_2_/CSMP adhesives increased by 5.1% (315.3 °C), 14.6% (302.1 °C), and 0.5% (333.1 °C), respectively (Figure 6g–i), showing that the single-network structure formed by hydrogen bond improved the thermal stability of the adhesive. Compared with the single-network, the double-bond network structure contained covalent bonds showed higher thermal stability. The thermal decomposition temperature of HPMP, SBMP, and CSMP adhesives increased by 5.8% (317.2 °C), 17.8% (310.5 °C), and 0.9% (334.5 °C). These results showed that the double-network synergy of hydrogen bonds and covalent bonds could greatly improve the thermal stability of the adhesive. Among them, CSMP adhesive showed the highest thermal decomposition temperature because of the densest double network. The thermal decomposition temperature of SBPM adhesive was lower than that of HPMP adhesive, which might be due to the low protein content.

### 3.2. Water Resistance and Bonding Strength Test

**Figure 7:**
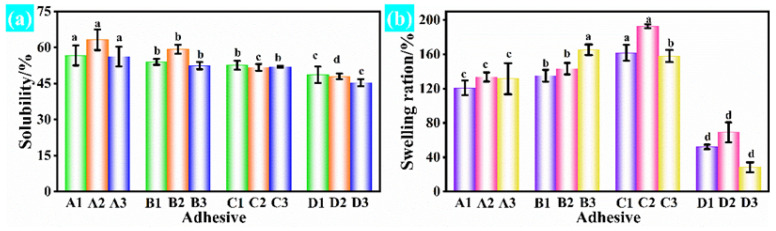
(**a**) Solubility; (**b**) swelling ration of A1-pure HPMP, B1-SDS/HPMP, C1-SDS/nSiO_2_/HPMP, D1-HPMP, A2-pure SBMP, B2-SDS/SBMP, C2-SDS/nSiO_2_/SBMP, D2-SBMP, A3-pure CSMP, B3-SDS/CSMP, C3-SDS/nSiO_2_/CSMP, and D3-CSMP adhesives.

Figure 7a,b showed the solubility and swelling rate of the different adhesives, respectively. Due to the high hydrophilicity of plant proteins, the interaction between proteins and wood (mechanical interlocking and hydrogen bond) was easy to break under wet conditions, resulting in poor water resistance. Therefore, the water resistance of adhesive played an important role in the bonding performance of adhesive [30]. The solubility of pure HPMP, SBMP, and CSMP adhesives were 56.7%, 63.2%, and 56.2%, respectively, and the swelling rates were 120.8%, 133.6%, and 131.5%, respectively. After SDS denaturation, the solubility of SDS/HPMP, SDS/SBMP, and SDS/CSMP adhesives decreased to 54%, 59.2%, and 52.4% due to the exposure of hydrophobic groups in the protein. The swelling rate showed an increasing trend, which was attributed to the changes of viscosity and contact angle. The hydrogen bond network formed by SiO_2_ with peanut, soybean, and cottonseed protein improved the water resistance, so the solubility of the SDS/nSiO_2_/HPMP, SDS/nSiO_2_/SBMP, and SDS/nSiO_2_/CSMP adhesives were reduced by 7.2% (56.7%), 16.8% (63.2%), and 7.5% (56.2%), respectively. The swelling rates of the SDS/nSiO_2_/HPMP, SDS/nSiO_2_/SBMP, and SDS/nSiO_2_/CSMP adhesives were further increased to 161.7%, 192.8%, and 157.9%, which was attributed to the improvement of elongation by nSiO_2_. In general, reducing the number of hydrophilic groups and increasing the crosslinking density were conducive to enhance the water resistance of the adhesive [31]. Therefore, the double-network structure formed after the introduction of PAE had better solubility and swelling rate. The results showed that CSMP adhesive had the best water resistance, and the water resistance of SBMP adhesive was slightly better than HPMP adhesive.

**Figure 8:**
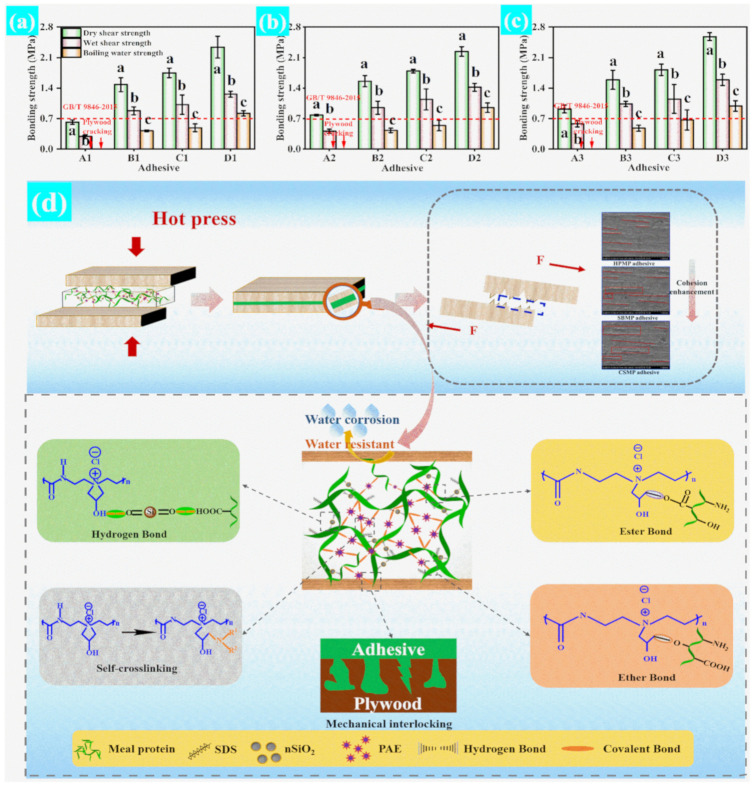
(**a**–**c**) Dry shear strength, wet shear strength, and boiling water strength of A1-pure HPMP, B1-SDS/HPMP, C1-SDS/nSiO_2_/HPMP, D1-HPMP adhesives, A2-pure SBMP, B2-SDS/SBMP, C2-SDS/nSiO_2_/SBMP, D2-SBMP adhesives, A3-pure CSMP, B3-SDS/CSMP, C3-SDS/nSiO_2_/CSMP, and D3-CSMP adhesives. (**d**) Enhancement mechanism of HPMP, SBMP, and CSMP adhesives.

Figure 8a–c shows the BWS, WSS, and DSS of different adhesives. Pure HPMP, SBMP, and CSMP adhesives would crack in boiling water. The WSS in warm water were 0.29 MPa, 0.41 MPa, and 0.58 MPa, and the DSS were 0.62 MPa, 0.78 MPa, and 0.92 MPa, respectively. After the introduction of SDS, the boiling water resistance of SDS/HPMP, SDS/SBMP, and SDS/CSMP adhesives were enhanced, and the BWS in boiling water were 0.42 MPa, 0.43 MPa, and 0.48 MPa. The WSS was significantly increased by 203.4% (0.88 MPa), 131.7% (0.95 MPa), and 79.3% (1.04 MPa), and the DSS was increased by 138.7% (1.48 MPa), 100% (1.56 MPa), and 72.8% (1.59 MPa), respectively. It was attributed to the following: (1) the protein peptide chain was destroyed, and more hydrophobic groups were exposed to enhance the hydrophobicity. (2) The increase of viscosity is conducive to the longitudinal penetration of the adhesive. The BWS of SDS/nSiO_2_/HPMP, SDS/nSiO_2_/SBMP, and SDS/nSiO_2_/CSMP adhesives increased to 0.55 MPa, 0.64 MPa, and 0.67 MPa; the WSS increased by 251.7% (1.02 MPa), 178.0% (1.14 MPa), and 98.3% (1.15 MPa); and the DSS increased by 182.3% (1.75 MPa), 129.5% (1.79 MPa), and 97.8% (1.82 MPa), which was attributed to the hydrogen bond network formed between protein and nSiO_2_. The BWS of HPMP, SBMP, and CSMP adhesives increased to 0.82 MPa, 0.95 MPa, and 0.99 MPa, which all satisfied the bonding strength of type I plywood standard (≥0.70 MPa). The WSS increased by 334.5% (1.26 MPa), 246.3% (1.42 MPa), and 174.1% (1.59 MPa), and the DSS also increased by 277.4% (2.34 MPa), 187.2% (2.24 MPa), and 202.2% (2.78 MPa), respectively. There were three reasons: (1) PAE optimized the viscosity of the adhesive, which was conducive to the extension and penetration on the wood surface, and formed more mechanical glue nails. (2) The hydrogen bond absorbed and dissipated the force in the fracture process to ensure sufficient toughness of the adhesive [19,32]. (3) The synergistic effect of this double network improved the mechanical properties of the adhesive. The SEM images (Figure 8d) of HPMP, SBMP, and CSMP adhesive plywood samples showed different degrees of failure modes (wood failure and fiber breakage) after bonding strength measurement. There were fibers on surfaces, and it showed that the double-network structure had good effects on three different plant protein adhesives [33]. The CSMP adhesive-bonded plywood had the roughest surface and the most wood fiber, the surface fiber of HPMP adhesive-bonded plywood was the least, which was the same as the BWS of the three adhesives (CSMP adhesive was the highest, SBMP adhesive was the second, and HPMP adhesive was the lowest). The mechanism for improving the bonding strength is shown in Figure 8d.

Table 2 shows the bonding strength (BWS, WSS, and DSS) comparison between the adhesives developed in this study and the previously reported eco-friendly adhesives. It could be seen that the BWS, WSS, and DSS of HPMP, SBMP, and CSMP adhesives were significantly higher than that of other eco-friendly adhesives. It also proved that this design had good universality, which provides a new theory for preparing protein adhesive with high bonding strength and strong water resistance.

### 3.3. Physical Property

**Figure 9:**
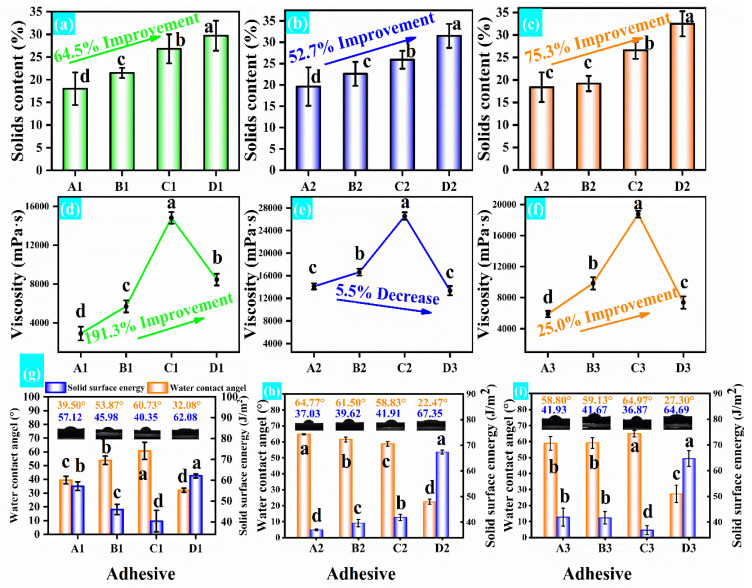
The (**a**–**c**) solids content, (**d**–**f**) viscosity, (**g**–**i**) water contact angle, and solid surface energy of A1-pure HPMP, B1-SDS/HPMP, C1-SDS/nSiO_2_/HPMP, D1-HPMP, A2-pure SBMP, B2-SDS/SBMP, C2-SDS/nSiO_2_/SBMP, D2-SBMP, A3-pure CSMP, B3-SDS/CSMP, C3-SDS/nSiO_2_/CSMP, and D3-CSMP adhesives.

The solids content was an important physical property of wood adhesive. Low solids content means high moisture content; the adhesive would penetrate laterally, and bubbles would appear during hot pressing. If the solid content is too high, it would not be conducive to the longitudinal penetration of the adhesive to the wood board and thus affect the plywood quality [15]. Therefore, the proper solids content range (20–35%) of adhesive is vital to improve the mechanical properties and plywood quality. As shown in Figure 9a–c, the solids content of the three pure adhesives (pure HPMP, SBMP, and CSMP adhesives) was very low (18.0%, 19.6%, and, 18.4%). After the addition of nSiO_2_, the solids content increased significantly by 48.9%, 60.7% and 44.6%, which was attributed to physical filling. The solids content of the three adhesives (HPMP, SBMP, and CSMP adhesive) showed the same change trend, increasing by 65.0%, 60.7%, and 76.6% respectively, and remained in the normal range.

Viscosity is an important parameter referring to handling and surface wetting ability of adhesives [37]. The viscosity of pure HPMP and CSMP adhesives were lower than the normal range, while the viscosity of pure soybean meal adhesive was within the normal range (Figure 9d–f), but the water resistance was poor. The SDS could destroy the secondary structure of protein and reduce the axial spacing between molecules so as to increase the friction [38]. Therefore, the viscosity of SDS/HPMP, SDS/SBMP, and SDS/CSMP adhesives was increased by 96.1%, 17.8%, and 67.0% respectively. After the addition of nSiO_2_, the viscosity of SDS/nSiO_2_/HPMP, SDS/nSiO_2_/SBMP, and SDS/nSiO_2_/CSMP adhesives showed different degrees of improvement again because nSiO_2_ could combine with protein molecules to form hydrogen bonds and inhibit its fluidity [39], but the viscosity was too large to be applied. After PAE crosslinking, the viscosity of the three adhesives (HPMP, SBMP, and CSMP adhesives) decreased significantly and remained in the normal range. There were two reasons: (1) The positively charged groups of PAE formed electrostatic interaction with charged protein chains, resulting in the reduction of attraction and repulsion between surrounding molecules, thus reducing the viscosity [25]. (2) The viscosity of PAE itself was lower than adhesive. In the process of crosslinking, PAE could be embedded into molecules and act as a “lubricant” [40]. Thus, the coatability and permeability of the adhesive were improved. In the process of practical application, we found that SBMP adhesive had the best applicating performance, and the applicated effect of HPMP adhesive was slightly better than that of CSMP adhesive; it was related to the natural structure of protein. This was also an important reason why SBMP adhesive could be sold in plywood enterprises, and the viscosity of HPMP adhesive and CSMP adhesive needs to be further optimized.

Water contact angle (WCA) is a significant parameter of an adhesive’s affinity for an adherent, where smaller WCA indicates better wettability [41]. There was a negative correlation between the solid surface energy and the WCA (Figure 9g–i). In fact, adhesives with small WCA and high solid surface energy are easy to spread onto a substrate surface. The pure SBMP adhesive exhibited high WCA (64.77°) and poor solid surface energy (37.03 J/m^2^) owing to its high molecular mass. On the contrary, the solid surface energy of HPM adhesive was the highest (57.12 J/m^2^), while CSMP adhesive was the second (41.93 J/m^2^). The WCA of three adhesives increased after adding SDS and nSiO_2_, while the solid surface energy decreased, which was related to their increasing viscosity. The WCA of HPMP, SBMP, and CSMP adhesives decreased significantly, and the solid surface energy increased significantly, indicating that PAE effectively improved the wettability of adhesives and was conducive to transverse spreading on the wood surface.

### 3.4. Microscopic Morphology Observation

**Figure 10:**
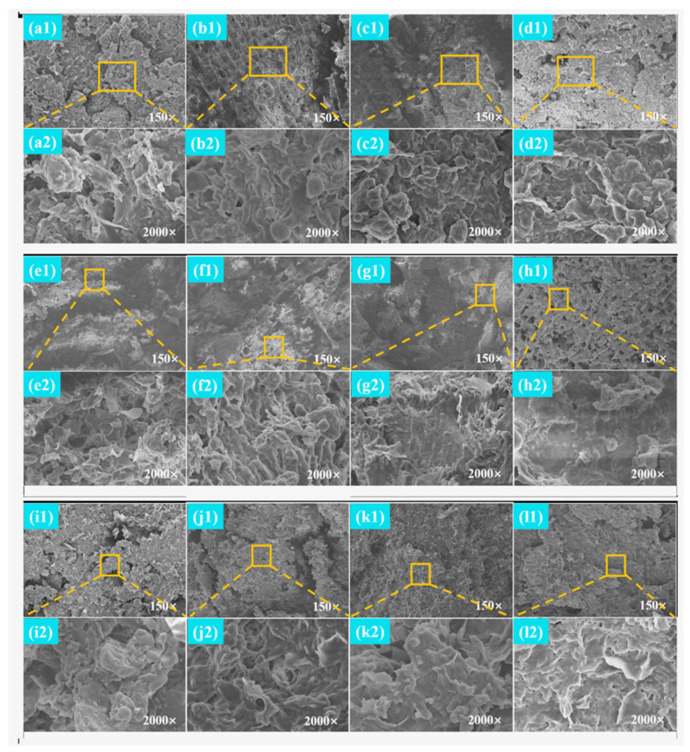
SEM images of (**a1**,**a2**) A1-pure HPMP, (**b1**,**b2**) B1-SDS/HPMP, (**c1**,**c2**) C1-SDS/nSiO_2_/HPMP, (**d1**,**d2**) D1-HPMP adhesives; SEM images of (**e1**,**e2**) B1-pure SBMP, (**f1**,**f2**) B2-SDS/SBMP, (**g1**,**g2**) C2-SDS/nSiO_2_/SBMP, (**h1**,**h2**) and D2-SBMP adhesives; SEM images of (**i1**,**i2**) A3-pure CSMP, (**j1**,**j2**) B3-SDS/CSMP, (**k1**,**k2**) C3-SDS/nSiO_2_/CSMP, (**l1**,**l2**) and D3-CSMP adhesives.

Figure 9 provides the microcosmic SEM images of different cured adhesives. There were many irregular holes and cracks on the surface of the three adhesives (pure HPMP, SBMP, and CSMP adhesives), which means that water was easy to invade (Figure 10a1,a2,e1,e2,i1,i2), resulting in poor water resistance [42]. However, more cracks appeared on the surface of SDS/HPMP, SDS/SBMP, and SDS/CSMP adhesives (Figure 10b1,b2,f1,f2,j1,j2) because SDS destroyed the secondary structure of protein. Although the cracks were more regular, they showed brittle fracture. The number of large holes in SDS/nSiO_2_/HPMP, SDS/nSiO_2_/SBMP, and SDS/nSiO_2_/CSMP adhesives decreased significantly (Figure 10c1,g1,k1). After 2000× magnification (Figure 10c2,g2,k2), it could be seen that the number of cracks on the surface was significantly reduced, but there were ups and downs of folds. It was attributed to the formation of the first level of the network structure by hydrogen bonding between nSiO_2_ and the active group of the protein. The holes and cracks of HPMP, SBMP, and CSMP adhesives were further reduced (Figure 10d1,h1,l1). It could be clearly observed that the surface became denser, which was due to the synergistic effect of the double network of hydrogen bonds and covalent bonds to form a certain network structure. The above results showed that the double-network structure could significantly improve the water resistance of the three different adhesives. After the comparison of the three adhesives (HPMP, SBMP, and CSMP adhesives), it was found that the surface of CSMP adhesive was the densest, indicating that water resistance was the best, while the surface of HPMP adhesive was the most wrinkled, which also supported the test results of bonding strength and water resistance.

### 3.5. Crack Observation

**Figure 11:**
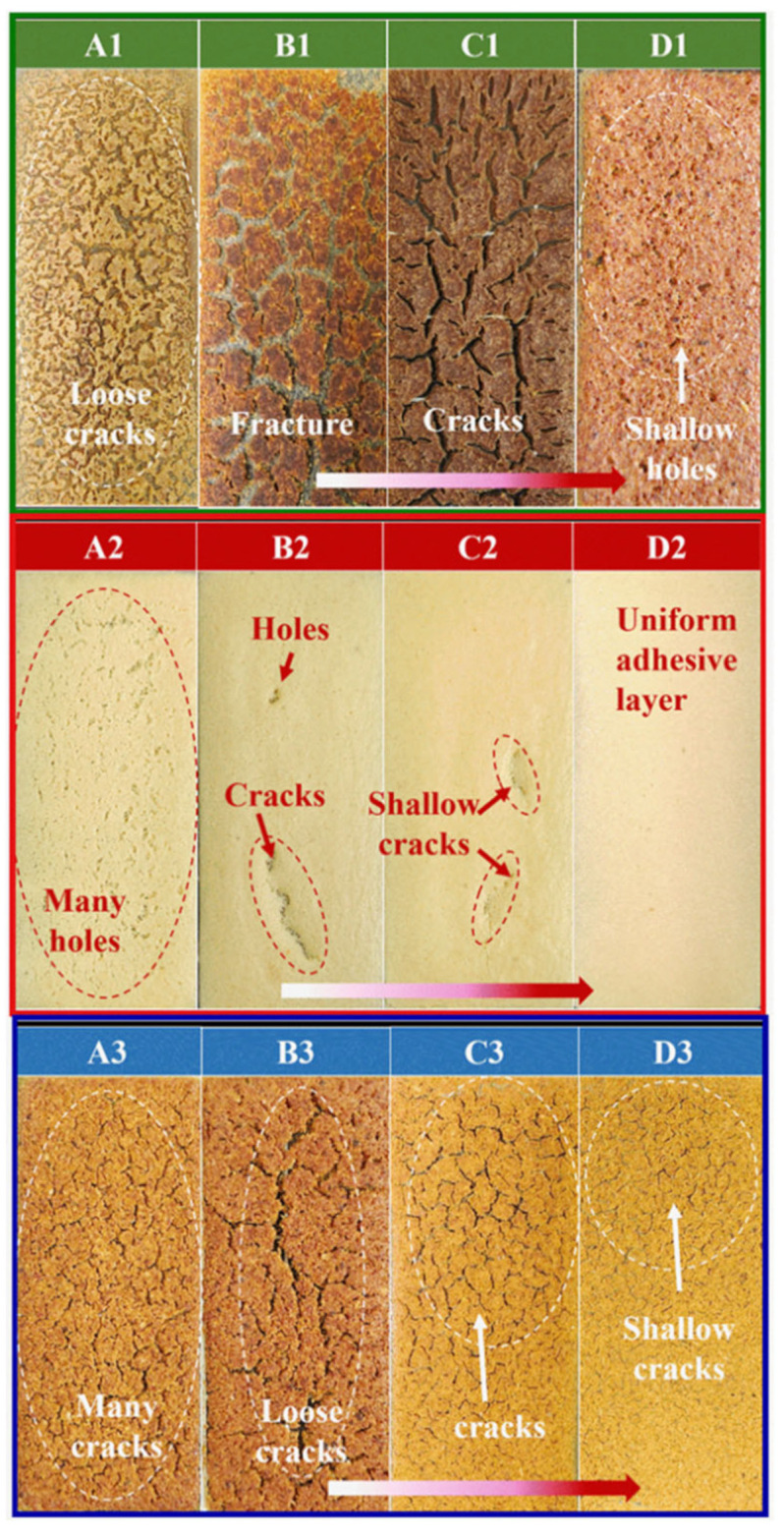
Cracks on the surface of A1-pure HPMP, B1-SDS/HPMP, C1-SDS/nSiO_2_/HPMP, D1-HPMP, A2-pure SBMP, B2-SDS/SBMP, C2-SDS/nSiO_2_/SBMP, D2-SBMP, A3-pure CSMP, B3-SDS/CSMP, C3-SDS/nSiO_2_/CSMP, and D3-CSMP adhesives.

The size and thickness of surface cracks of different cured adhesives could be compared through crack observation (Figure 11), evaluating the toughness and heat resistance of different adhesives [43]. There were many large holes and cracks on the surface of cured pure HPMP, SBMP, and CSMP adhesive, and the adhesives were loose and fragile, which was reflected their inherent brittleness. Although the holes of SDS/SBMP adhesive decreased, they showed larger cracks. The SDS/HPMP and SDS/CSMP adhesives also showed larger cracks because SDS destroyed the secondary structure of protein, reduced their cohesion, and increased the brittleness of the adhesive [44]. After the introduction of SiO_2_, there were only a few cracks in the surface of the cured SDS/nSiO_2_/SBMP adhesive, and the surface of cured SDS/nSiO_2_/HPMP and SDS/nSiO_2_/CSMP adhesives were closer, but there were still many uneven cracks, indicating that the cohesion provided by hydrogen bond was not enough to maintain toughness. The surface of the cured SBMP adhesive looked smoother because the soybean protein adhesive had high viscosity and covered the holes. There were still a few holes on the surface of cured HPMP adhesive, while the cracks and holes on the surface of cured CSMP adhesive were further reduced and dispersed. This was due to the dissipation of the internal stress by the double-network structure composed of covalent bonds and hydrogen bonds before it played its role.

### 3.6. Current Problems and Future Prospects

Compared with trialdehyde adhesive, plant protein adhesive could solve the problem of formaldehyde release. However, due to the limitation of raw materials and preparation technology, there are still the following four problems: (1) Plant protein adhesive is prone to mildew. (2) The bonding strength is unstable. (3) The initial adhesion of plant protein adhesives is poor. (4) Plant protein adhesives need crosslinking agents, so they are costly. This is also the direction of future research. It is believed that with the in-depth research of plant protein adhesives and the gradual solution of practical application problems, as one of the most environmentally friendly natural biomass adhesives with development potential, plant protein adhesives will be widely applied in the production of wood-based panels.

China is a big consumer of grain and oil. For agri-food business operators, hot-pressed peanut meal containing *Aspergillus flavus* and cottonseed meal containing gossypol are wastes. Their annual output exceeds 10 million tons, which is an endless stream of green, raw materials. However, they cannot be processed into food or feed, so they can provide raw materials to adhesives or plywood companies to make protein adhesives, solving the waste recycling problem and generating additional income.

## 4. Conclusions

This study reported a low-cost, double-network construction theory, which was applied to high-performance HPMP, SBMP and CSMP adhesives. Under the synergistic action of hydrogen bonds and covalent bonds, the BWS of HPMP, SBMP, and CSMP adhesives exceeded the bonding strength requirement of type I plywood (0.7 MPa) by 17.1%, 35.7%, and 41.4%. The CSMP adhesive showed better water resistance and bonding strength than SBMP adhesive, and HPMP adhesive was also close to SBMP adhesive. Importantly, the cost of HPM and CSM was far lower than that of SBM, which helped to solve the problem of high dependence on soybean meal and provided an important theory basis for the high-value-added utilization of different kinds of plant meal.

## Figures and Tables

**Table 1:** Compositions of the HPMP-, SBMP-, and CSMP-based adhesive.

Adhesive	HPM (g)	SBM (g)	CSM (g)	Water (g)	SDS (g)	nSiO_2_	PAE (g)
A1	40			120			
B1	40			120	1.28		
C1	40			120	1.28	0.8	
D1	40			120	1.28	0.8	34
A2		40		120			
B2		40		120	1.28		
C2		40		120	1.28	0.8	
D2		40		120	1.28	0.8	34
A3			40	120			
B3			40	120	1.28		
C3			40	120	1.28	0.8	
D3			40	120	1.28	0.8	34

A1, pure HPMP adhesive; B1, SDS/HPMP adhesive; C1, SDS/nSiO_2_ HPMP adhesive; D1, HPMP adhesive; A2, pure SBMP adhesive; B2, SDS/SBMP adhesive; C2, SDS/nSiO_2_ SBMP adhesive; D2, SBMP adhesive; A3, pure CSMP adhesive; B3, SDS/CSMP adhesive; C3, SDS/nSiO_2_ CSMP adhesive; D3, CSMP adhesive.

**Table 2:** Comparison of matrix, the boiling water strength, wet shear strength, and dry shear strength of different adhesives.

Matrix	Adhesive	Dry Shear Strength/MPa	Wet Shear Strength/MPa	Boiling Water Strength/MPa	Testing Standards	Refs
Peanut meal	HPMP	2.34	1.26	0.82	GB/T 9846–2015	This work
	Peanut meal/U/ECH	1.79	0.92	-	GB/T 9846–2015	[10]
	HPM/SDS	-	1.05	-	GB/T 9846–2015	[34]
Soybean meal	SBMP	2.24	1.42	0.95	GB/T 9846–2015	This work
	AC@CNF-PA/SM-2	1.71	1.19	-	GB/T 9846–2015	[13]
	6-SM/BD/5%HPFC	2.04	1.05	-	GB/T 9846–2015	[22]
	SF/XSBRL	1.44	0.97		GB/T 9846–2015	[35]
	SP/WE-PAM	-	1.16	-	GB/T 9846–2015	[36]
Cottonseed meal	CSMP	2.58	1.59	0.99	GB/T 9846–2015	This work

## Data Availability

Not applicable.

## Supplementary Materials

The following supporting information can be downloaded at: https://www.mdpi.com/article/10.3390/foods11182839/s1, Figure S1: XPS spectra of a broad scan survey of (a) A1-pure HPMP, B1-SDS/HPMP, C1- SDS/nSiO_2_/HPMP, and D1-HPMP adhesives. (b) A2-pure SBMP, B2-SDS/SBMP, C2-SDS/nSiO_2_/SBMP, and D2-SBMP adhesives. (c) A3-pure CSMP, B3-SDS/CSMP, C3- SDS/nSiO_2_/CSMP, and D3-CSMP adhesives.

## References

[1] Chen H., Chen S., Fan D., Wang Y. (2021). Preparation and Characterization of a Robust, High Strength, and Mildew Resistant Fully Biobased Adhesive from Agro-Industrial Wastes. ACS Appl. Polym. Mater..

[2] Zajforoushan Moghaddam S., Qie R., Thormann E. (2022). Making Protein-Based Adhesives Water Resistant: Role of Protein Water Solubility, Galloyl Modification, and Complexation. ACS Appl. Polym. Mater..

[3] Chen X., Ji N., Li F., Qin Y., Wang Y., Xiong L., Sun Q. (2022). Dual Cross-Linked Starch–Borax Double Network Hydrogels with Tough and Self-Healing Properties. Foods.

[4] Fraga-Corral M., Otero P., Cassani L., Echave J., Garcia-Oliveira P., Carpena M., Chamorro F., Lourenço-Lopes C., Prieto M.A., Simal-Gandara J. (2021). Traditional Applications of Tannin Rich Extracts Supported by Scientific Data: Chemical Composition, Bioavailability and Bioaccessibility. Foods.

[5] Tribot A., Amer G., Abdou Alio M., de Baynast H., Delattre C., Pons A., Mathias J.-D., Callois J.-M., Vial C., Michaud P. (2019). Wood-lignin: Supply, extraction processes and use as bio-based material. Eur. Polym. J..

[6] Yin H., Zhang E., Zhu Z., Han L., Zheng P., Zeng H., Chen N. (2021). Soy-Based Adhesives Functionalized with Pressure-Responsive Crosslinker Microcapsules for Enhanced Wet Adhesion. ACS Appl. Polym. Mater..

[7] Li Z., Zhao S., Wang Z., Zhang S., Li J. (2020). Biomimetic water-in-oil water/pMDI emulsion as an excellent ecofriendly adhesive for bonding wood-based composites. J. Hazard. Mater..

[8] Zheng P., Chen N., Mahfuzul Islam S.M., Ju L.-K., Liu J., Zhou J., Chen L., Zeng H., Lin Q. (2019). Development of self-cross-linked soy adhesive by enzyme complex from aspergillus niger for production of all-biomass composite materials. ACS Sustain. Chem. Eng..

[9] Li X., Li J., Luo J., Li K., Gao Q., Li J. (2018). A novel eco-friendly blood meal-based bio-adhesive: Preparation and performance. J. Polym. Environ..

[10] Chen C., Chen F., Liu B., Du Y., Liu K. (2019). Peanut meal-based wood adhesives enhanced by urea and epichlorohydrin. R. Soc. Open Sci..

[11] Khoo S.C., Peng W.X., Yang Y., Ge S.B., Soon C.F., Ma N.L., Sonne C. (2020). Development of formaldehyde-free bio-board produced from mushroom mycelium and substrate waste. J. Hazard. Mater..

[12] Krüger J.M., Brner H.G. (2021). Accessing the next generation of synthetic mussel-glue polymers via mussel-inspired polymerization. Angew. Chem. Int. Ed..

[13] Jin S., Li K., Zhang X., Gao Q., Zeng L., Shi S.Q., Li J. (2020). Phytic acid-assisted fabrication for soybean meal/nanofiber composite adhesive via bioinspired chelation reinforcement strategy. J. Hazard. Mater..

[14] Cheng H.N., Ford C., Dowd M.K., He Z. (2016). Use of additives to enhance the properties of cottonseed protein as wood adhesives. Int. J. Adhes. Adhes..

[15] Gu W., Liu X., Ye Q., Gao Q., Gong S., Li J., Shi S.Q. (2020). Bio-inspired co-deposition strategy of aramid fibers to improve performance of soy protein isolate-based adhesive. Ind. Crops Prod..

[16] Chen H., Liu Y., Ren B., Zhang Y., Ma J., Xu L., Chen Q., Zheng J. (2017). Super bulk and interfacial toughness of physically crosslinked double-network hydrogels. Adv. Funct. Mater..

[17] Li X., Tang C., Liu D., Yuan Z., Hung H.-C., Luozhong S., Gu W., Wu K., Jiang S. (2021). High-strength and nonfouling zwitterionic triple-network hydrogel in saline environments. Adv. Mater..

[18] Yang Z., Zhang X., Liu X., Guan X., Zhang C., Niu Y. (2017). Polyglycerol-based organic-inorganic hybrid adhesive with high early strength. Mater. Des..

[19] Xu Y., Han Y., Chen M., Luo J., Shi S.Q., Li J., Gao Q. (2021). Constructing a triple network structure to prepare strong, tough, and mildew resistant soy protein adhesive. Compos. B Eng..

[20] Li X., Deng Y., Lai J., Zhao G., Dong S. (2020). Tough, long-eerm, water-resistant, and underwater adhesion of low-molecular-weight supramolecular adhesives. J. Am. Chem. Soc..

[21] Qu Y., Guo Q., Li T., Zhang Y., Gao Q., Liu H., Wang Q. (2021). A novel environmentally friendly hot-pressed peanut meal protein adhesive. J. Clean. Prod..

[22] Xu Y., Huang X., Zhang Y., Liu Z., Luo J., Li J., Li J., Gao Q. (2021). A high bonding performance and antibacterial soybean meal adhesive with maillard reaction based cross-linked structure. Compos. B Eng..

[23] (2015). China National Standard Plywood for General Use.

[24] Costa S.M., Ferreira D.P., Ferreira A., Vaz F., Fangueiro R. (2018). Multifunctional flax fibres based on the combined effect of silver and zinc oxide (Ag/ZnO) nanostructures. Nanomaterials.

[25] Liu H., Bean S., Sun X.S. (2018). Camelina protein adhesives enhanced by polyelectrolyte interaction for plywood applications. Ind. Crops Prod..

[26] Zhou Y., Zeng G., Zhang F., Luo J., Li K., Li X., Li J., Fang Z. (2022). High strength and flame retardant soybean polysaccharide-based wood adhesive produced by borate chemistry and crosslinking strategy. Eur. Polym. J..

[27] Zhao S., Wang Z., Li Z., Li L., Zhang S. (2019). Core-shell nanohybrid elastomer based on co-deposition strategy to improve performance of soy protein adhesive. ACS Appl. Mater. Interfaces.

[28] Zhao S., Wang Z., Kang H., Li J., Zhang S., Han C., Huang A. (2018). Fully bio-based soybean adhesive in situ cross-linked by interactive network skeleton from plant oil-anchored fiber. Ind. Crops Prod..

[29] Li J., Xiao F., Amirkhanian S.N. (2019). Storage, fatigue and low temperature characteristics of plasma treated rubberized binders. Constr. Build. Mater..

[30] Liu M., Wang Y., Wu Y., He Z., Wan H. (2018). “Greener” adhesives composed of urea-formaldehyde resin and cottonseed meal for wood-based composites. J. Clean. Prod..

[31] Xu H., Yang M., Hou X., Li W., Su X., Yang Y. (2016). Industrial trial of high-quality all green sizes composed of soy-derived protein and glycerol. J. Clean. Prod..

[32] Mazzotta M.G., Putnam A.A., North M.A., Wilker J.J. (2020). Weak bonds in a biomimetic adhesive enhance toughness and performance. J. Am. Chem. Soc..

[33] Zhao S., Pang H., Li Z., Wang Z., Kang H., Zhang W., Zhang S., Li J., Li L. (2021). Polyurethane as high-functionality crosslinker for constructing thermally driven dual-crosslinking plant protein adhesion system with integrated strength and ductility. Chem. Eng. J..

[34] Chen Y., Shi A., Wang Q. (2018). Peanut meal as plywood adhesives: Preparation and characterization. J. Adhes. Sci. Technol..

[35] Li H., Kang H., Zhang W., Zhang S., Li J. (2016). Physicochemical properties of modified soybean-flour adhesives enhanced by carboxylated styrene-butadiene rubber latex. Int. J. Adhes. Adhes..

[36] Wang Z., Chen Y., Chen S., Chu F., Zhang R., Wang Y., Fan D. (2019). Preparation and characterization of a soy protein based bio-adhesive crosslinked by waterborne epoxy resin and polyacrylamide. RSC Adv..

[37] Cheng H.N., Ford C., Dowd M.K., He Z. (2016). Soy and cottonseed protein blends as wood adhesives. Ind. Crops Prod..

[38] Wei Y., Yao J., Shao Z., Chen X. (2020). Water-resistant zein-based adhesives. ACS Sustain. Chem. Eng..

[39] Li H., Li C., Gao Q., Zhang S., Li J. (2014). Properties of soybean-flour-based adhesives enhanced by attapulgite and glycerol polyglycidyl ether. Ind. Crops Prod..

[40] Yuan C., Chen M., Luo J., Li X., Gao Q., Li J. (2017). A novel water-based process produces eco-friendly bio-adhesive made from green cross-linked soybean soluble polysaccharide and soy protein. Carbohydr. Polym..

[41] Wang Z., Zhao S., Zhang W., Qi C., Zhang S., Li J. (2019). Bio-inspired cellulose nanofiber-reinforced soy protein resin adhesives with dopamine-induced codeposition of “water-resistant” interphases. Appl. Surf. Sci..

[42] Zhang J., Zhang M., Zhang Y., Shi S.Q., Gao Q. (2020). Improving bond performance and reducing cross-linker dosage for soy flour adhesives inspired by spider silk. ACS Sustain. Chem. Eng..

[43] Li X., Xia C., Li J., Zhou X. (2020). Design and build an elastic crosslinked network to strengthen and toughen soybean-meal based bioadhesive using organo-sepiolite and greener crosslinker triglycidylamine. Polym. Test..

[44] Zhang Y., Shi R., Xu Y., Chen M., Zhang J., Gao Q., Li J. (2020). Developing a stable high-performance soybean meal-based adhesive using a simple high-pressure homogenization technology. J. Clean. Prod..

