# Supplementary material for: Preparation and Characterization of Plant Protein Adhesives with Strong Bonding Strength and Water Resistance
Source: Foods. 2022 Sep 14;11(18):2839. doi: 10.3390/foods11182839 (PMC9497928; doi:10.3390/foods11182839)
Supplement: Supplementary file 1 [file foods-11-02839-s001.zip › foods-1911562-Supplementary Material.pdf]

# Preparation and Characterization of Plant Protein Adhesives with Strong Bonding Strength and Water Resistance

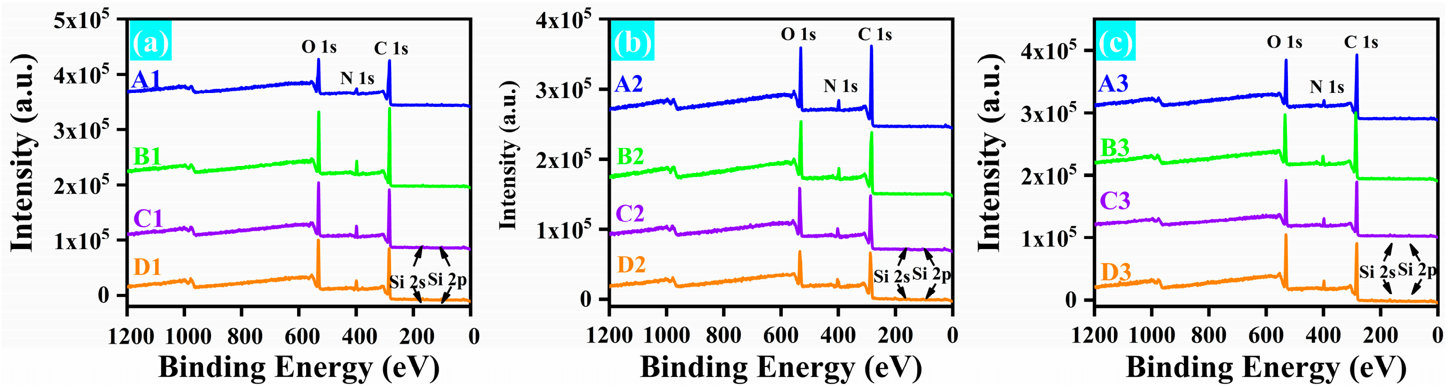


**Figure S1.** XPS spectra of a broad scan survey of (a) A1-pure HPMP, B1-SDS/HPMP, C1- SDS/nSiO$_2$/HPMP, and D1-HPMP adhesives. (b) A2-pure SBMP, B2-SDS/SBMP, C2-SDS/nSiO$_2$/SBMP, and D2-SBMP adhesives. (c) A3-pure CSMP, B3-SDS/CSMP, C3- SDS/nSiO$_2$/CSMP, and D3-CSMP adhesives.
